# Items

**Published:** 1878-09

**Authors:** 


### Items.

—Boston Medical and Surgical Journal for August 15th, announces the sudden death of Dr. George W. Gay, of that city, from Cardiac disease of long standing. He was a frequent contributor to Medical Literature, and was well known and highly respected in the profession and by all who knew him.

—At the Fourth Annual Meeting of the American Neurological Association, held in June in New York City, a prize of two hundred and fifty dollars offered by Wm. A. Hammond, M. D., was awarded to E. C. Spitzka, M. D., of New York for an essay on the Physiological Action of Strychnia on the Brain, Spinal Cord and Nerves. Dr. Hammond renews his prize this year, the subject and conditions, not yet being stated. At the same meeting, Dr. J. J. Putnam, of Boston, read a paper upon Observations on the Intra-Ocular Circulation, made by himself and Dr. O. F. Wadsworth. After experimenting upon themselves with atropine, nitrite of amyl, pressure upon the carotids and jugular veins, and making tracings from the surface of the brain of a man who had received fractures of the skull, they arrived at the following conclusion as reported in the *Medical Record:* that mechanical causes did not readily, if at all, induce appreciable arterial congestion of the fundus oculi, and the degree of nervous fulness so produced was not such as to justify, if seen in an eye examined for the first time, the belief that it was pathological. They were therefore led to the doubt whether such condition as congestion of the retina, unassociated with inflammation of a greater or less degree, in which case the term congestion would be, properly speaking, a misnomer, was ever met with.

—Mortuary.—The death of Carl Rokitansky is recently announced by telegram from Vienna. He was seventy-four years of age, born in Bohemia, was early connected with the Institute of Pathological Anatomy in Vienna, held various positions of honor, and in 1850 was made Rector of the University of Vienna. His excellent work upon Pathological Anatomy in four volumes was published in 1842, at which time he was said to have made upwards of thirty thousand post-mortem examinations. This work was republished by the Sydenham Society. He was chief of the Viennese school of Pathology.

—Henry A. Martin, M. D., of Boston Highlands, uses in varicose ulcers of the leg, an Esmarch bandage made of rubber sheeting. He recommends it also in diseases and injuries of joints, housemaid's knee and other affections. The bandage is secured to the limb by a bit of tape fastened at the end of the bandage, and girt about the extremity. —Professor W. H. Van Buren, M.D., of New York City, contributed an interesting and valuable article on Extirpation of the Rectum, by Volkmann's method, for cancer, with cases, in the Medical Record for July thirteenth, —Dr. Martin, of Berlin, reported to the Surgical Section of the British Medical Association, at its last meeting, a case of removal of the spleen. The patient was a feeble, hunchback woman thirty-one years of age, suffering from uterine disturbances which appeared to be aggravated by the spleen getting down into the pelvis. The operation was done under antiseptic precautions, the pedicle was long, and the wound healed by first intention. The subsequent history of the case is not given. There had been nine cases of splenotomy previous to this, of which three recovered, one died of pyæmia, two of shock and three of hemorrhages.

—Several cases have been recorded of mortification of stumps following the use of Esmarch's bandage in amputation.—*Boston Journal.*

—The New York Dispensary makes use of the following:—Pulvis Sennæ Compositus. R. Pulvis Sennæ, Potass., Bitart., Sulphur Sublim., of each two ounces; Pulv. Zinziberis, two drachms, Mix. Dose—one teaspoonful. ——*New Remedies* gives the following formula for Elixir of Cinchona from Alkaloids:—R. Quinia Sulphate, eight grains; Cinchonia Sulphate, thirtytwo grains; Cinchonidia Sulphate, sixteen grains; Citric Acid, ten grains; Alcohol, (95 per cent.) two fluid ounces; Water, (boiling,) two fluid ounces. Dissolve the citric acid on two drachms of the water, to which add the alcohol, and to this solution add the quinia, cinchonia and cinchonidia, and then add the remainder of the water, which must be hot. Finally add the elixir. If desired this may be colored with tincture of cudbear and caramel.—Bromatic Dovers Powder is a very pleasant tasting preparation for children, which is used in Buffalo and made up thus, by A. R. Davidson, M. D ,—R. Pulv. Opii., Pulv. Ipecac, of each, one part, Pulv. Sacchari, six and one-half parts; Pulv. Cinnamoni, one and one-third parts; Pulv. Carophylli, one-sixth of a part. Mix thoroughly.——Goa powder or its active principle, chrysophanic acid is very highly recommended by Prof. Newman, Balmanno Squire, and others, in psoriasis. One drachm of chrysophanic acid is rubbed up with an ounce of hot lard and application of this ointment made at night to the affected part by means of a swab of flannel. This substance stains the skin and clothing, and therefore is of limited applicability. The dust of Goa powder is very irritating to the eyes of those engaged in preparing it.

—There were three deaths from yellow fever at the Brooklyn Navy Yard in July, but at present there are no cases there.

—"In the United States, with a population of 44,874,814, there are 62,383 doctors, or one doctor to every 600 persons. In France the population is 36,100,000, the physicians 19,902, one to every 1672 persons. In the German empire there are 13,686 doctors, and 41,060,695 inhabitants, one doctor to every 3,000. In the Austro-Hungarian empire, population 35,904,435, and 14,361 doctors,—one to every 2,500 persons. In Canada, with a population 3,57[illegible],577, there are 2,998 doctors, or one to every 1193 individuals."

—H. C. Lea has just issued a new edition of Gray's Anatomy. There have been three English revisions of this work since the last American edition was published, in 1870. The new issue is from the eighth enlarged and improved London edition and contains nearly one thousand pages, and five hundred and twenty-five illustrations. To it is appended also "Landmarks, Medical and Surgical," by Luther Holden, D. R., C. S., a small work recently issued here. The same publisher announces as in press:—the National Dispensatory, by Alfred Stillé, M. D., and John M. Maische, Ph. D.; a new edition of Barnes on Diseases of Women; and a Manual of Quantitative Analysis, by Alexander Classen. Fothergill's Antagonism of Medicines and a new edition of Bryant's Surgery will appear shortly. The following works are in course of preparation:—Cornil & Ranvier's Pathological Histology, translated with notes and additions, by E. O. Shakespeare, M. D., of Philadelphia; A System of Human Anatomy, in a quarto volume, by Harrison Allen, M. D., Professor of Comparative Anatomy and Physiology in the University of Pennsylvania; A Manual of the Diseases of Women, by James R. Chadwick, M. D.; also new editions of Playfair's Midwifery and Wells on the Eye.

—American Dermatological Association. The next annual meeting of the American Dermatological Association will be held at the Grand Union Hotel, Saratoga Springs, N. Y., on the 27th, 28th and 29th of August. Many important papers will be read. Regular practitioners are cordially invited.

R. W. Taylor, M. D., *Sec'y.*

—American Association for the Cure of Inebriates, will hold its Tenth Annual Meeting at Boston, Mass., Sept. 10th, 1878, in Union Hall. A very important meeting is anticipated.

T. D. Crothers, M. D., *Secretary.*

—In the year 1877, eleven hundred and forty-two horses were devoured in Marseilles. These figures indicate a slow but steady increase of the use of horse-flesh in that city.

